# Complete mitochondrial genomes and phylogenetic analysis of four Baikal endemic *Batrachocottus* species (Scorpaeniformes: Cottoidei)

**DOI:** 10.1080/23802359.2021.2013741

**Published:** 2022-02-03

**Authors:** Veronika Teterina, Bakhtiar Bogdanov, Sergei Kirilchik

**Affiliations:** Limnological Institute of Siberian Branch, Russian Academy of Sciences, Irkutsk, Russia

**Keywords:** Cottoidei, Baikal sculpins, *Batrachocottus*, mitochondrial genome, phylogeny

## Abstract

Baikal sculpins are the most species-rich and ecologically diverse group of fishes in the Lake. We analyzed complete mitochondrial genomes from four species of the endemic Baikal genus *Batrachocottus* (*B. baicalensis*, *B. multiradiatus*, *B. talievi*, and *B. nikolski*). Mitogenome sequences are 16,523–16,535 bp in length with a mitogenomic organization and gene arrangement identical to that of typical teleosts. Phylogenetic analysis using the Bayesian method positioned *B. baicalensis* outside the monophyletic clades of the genus *Batrachocottus*. *Batrachocottus multiradiatus* and *B. talievi* are sister species.

The endemic flock of Baikal sculpins (Cottoidei), according to the most recent calculation, includes 33 species (Sideleva 2003). Different species of this unique group are adapted to various depth habitats and diets, subdividing into three ecological groups: benthic, benthopelagic, and pelagic. The genus *Batrachocottus* (Big-headed sculpins) comprises four species: *B. baicalensis* (Dybowski, 1874), *B. nikolskii* (L. S. Berg, 1900), *B. multiradiatus* (L. S. Berg, 1907) and *B. talievi* (Sideleva, 1999). Molecular data confirmed the validity of two species within the genus (Kontula et al. 2003): *B. nikolskii* and *B. multiradiatus*. Here, we analyze for the first time molecular phylogeny involving the *B. baicalensis* and *B. talievi.* Among four *Batrachocottus* species only *B. baicalensis*, which mainly inhabited coastal shallow terrace zone, can be clearly defined morphologically. Three other species, which occur mostly in the deep-water zone, are often difficultly to identify because they form a «chain of forms» owing to the overlapped variation of morphometric characters (Bogdanov 2017). The questions about species distribution, their number, and population structure are still open. The mitochondrial genome-based phylogenetic analysis would improve our understanding of the evolutionary taxonomy and relationship between *Batrachocottus* species and other Cottoidei.

Here, we sequenced and analyzed the complete mitochondrial DNA sequences of four species of the genus *Batrachocottus*. All the samples were collected in Lake Baikal by nets and trawl (53°50′27′′N, 108°34′42′′E for *B. baicalensis,* 55°06′13′′N, 109°40′14′′E for *B. multiradiatus,* 54°48′30′′N, 109°28′81′′E for *B. nikolskii* and 53°41′68′′N, 108°38′41′′E for *B. talievi*). The total genomic DNA was isolated from fin clips by the conventional phenol-chloroform method (Sambrook et al. 1989).

Mitogenomes were generated using traditional Sanger sequencing on an ABI 3500 Genetic Analyzer (Applied Biosystems, USA). Primers were designed according to the conserved regions of the complete mitogenomes of other cottoid species. Sequence reads were assembled by CAP3 software (Huang and Madan 1999) and were aligned within the ClustalW program in BioEdit software. The lengths of mitogenomes are 16,523 bp for *B. baicalensis* with 47.67% GC content, 16,530 for *B. talievi* with 47.57% GC content, 16,532 for *B. multiradiatus* with 47.70% GC content, and 16,535 bp for *B. nikolskii* with 47.58% GC content. Mitochondrial genomes contain 2 ribosomal RNA genes, 22 transfer RNA genes, 13 protein-coding genes, and a control region with the gene order and codon usage identical to that of typical teleosts. The part of mitochondrial DNA of *B. multiradiatus* had a single nucleotide deletion in ND6, resulting in a frameshift. These can be mitochondrial heteroplasmy or nuclear insertion of mitochondrial pseudogenes (numts).

Phylogenetic analysis was performed using the sequences of 13 mitochondrial protein-coding genes of *Batrachocottus* and those of other Cottoidei species. Optimum evolutionary models of nucleotide substitutions for multilocus phylogeny were selected in PartitionFinder2 (Lanfear et al. 2017). The phylogenetic tree (Figure 1) was generated with the Bayesian inference method implemented in MrBayes v3.2.7 (Ronquist et al. 2012).

**Figure 1.**
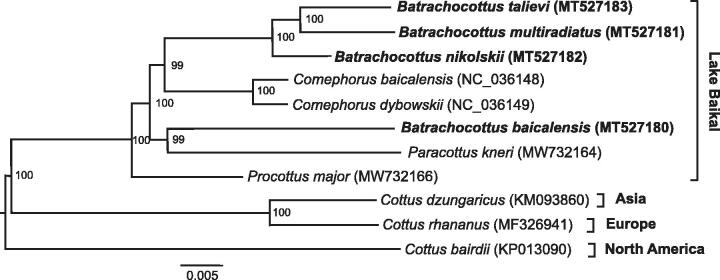
Multilocus Bayesian DNA phylogeny of the Cottoidei based on the nucleotide sequences of the 13 mitochondrial protein-coding genes. Posterior probabilities with 200,000 generations were shown next to nodes.

The monophyly of the genus Batrachocottus is not supported by the phylogenetic tree. Only three species, – *B. multiradiatus*, *B. nikolskii*, and *B. talievi*, formed monophyletic clade with bootstrap value = 100%, whereas *B. baicalensis* is outside the group. The phylogenetic analysis indicated that *B. multiradiatus* and *B. talievi* are sister species. This work confirmed the validity of four species, but there was a question about the belonging of the *B. baicalensis* to the genus Batrachocottus.

## Data Availability

The genome sequence data that support the findings of this study are openly available in GenBank (https://www.ncbi.nlm.nih.gov/) under the accession numbe*rs MT527180* (*B. baicalensis*), MT527181 (*B. multiradiatus*), MT527182 (*B. nikolskii*), and MT527183 (*B. talievi*). Specimens were deposited at Limnological Institute of the SB RAS, Irkutsk, Russia (http://lin.irk.ru , contact person: Veronika Teterina, email: veronika_t@inbox.ru) under the voucher number Bb20 for *B. baicalensis*, Bm2_O for *B. multiradiatus*, Bn316 for *B. nikolskii* and Bt268 for *B. talievi.*
